# Developing a Novel Digital Tool for Personalised Antipsychotic Prescribing in People Living With Dementia: The Views of Australian Clinicians

**DOI:** 10.1177/14713012251366757

**Published:** 2025-08-05

**Authors:** Timothy Josh D. Tan, Yun-Hee Jeon, Edward C. Y. Lau, Sarah N. Hilmer, Lee-Fay Low, Christine Y. Lu, Edwin C. K. Tan

**Affiliations:** 1 The University of Sydney School of Pharmacy, Faculty of Medicine and Health, 4334 The University of Sydney, Sydney, NSW, Australia; 2 Susan Wakil School of Nursing and Midwifery, Faculty of Medicine and Health, 4334 The University of Sydney, Sydney, NSW, Australia; 3 Kolling Institute, Faculty of Medicine and Health, The University of Sydney and the Northern Local Health District, Sydney, NSW, Australia; 4 The University of Sydney School of Health Sciences, Faculty of Medicine and Health, 4334 The University of Sydney, Sydney, NSW, Australia; 5 Department of Pharmacy, Royal North Shore Hospital, St Leonards, NSW, Australia

**Keywords:** dementia, antipsychotic agents, clinical decision support systems, shared decision-making, drug-related side effects and adverse reactions

## Abstract

Optimising antipsychotic prescribing in people living with dementia is important to manage symptoms and avoid adverse events. Clinical decision support tools that predict therapeutic response based on individual patient characteristics can help personalise prescribing and complement decision-making by prescribers. The aim of this study is to investigate the views of Australian prescribers on the development and use of a digital antipsychotic prescribing support tool in dementia. Thematic analysis was used to analyse the perspectives of Australian prescribers on using a digital prescribing support tool in dementia. Semi-structured, individual interviews were conducted with a sample of 14 clinicians. Themes were organised according to topic areas about the development and use of the tool. Clinicians expressed that the tool could assist in *identifying risk*, allowing prescribers to be more cautious with antipsychotic prescribing. The tool could promote *informed decision-making* by assisting prescribers to consider more factors prior to prescribing whilst serving as an educational tool to aid shared decision-making with patients and carers. Though there were benefits, clinicians raised that there are *complexities of antipsychotic prescribing,* as the tool may not account for situational need, where benefits may outweigh risks. Some clinicians expressed *potential concerns with technology-based tools*, where some prescribers may void their clinical judgement and over-rely on the tool. Some clinicians highlighted younger practitioners, general practitioners, nurses and pharmacists as *potential users* who could benefit from its use. Clinicians posed *suggestions for development*, including accessibility through an app, updating data as evidence and guidelines change, and prompts to aid decision-making. This study identified several considerations on the implementation of the tool in clinical practice. Perspectives raised by clinicians should be considered in the tool’s future development.

## Introduction

Dementia is a growing public health concern, affecting more than 55 million people worldwide with an annual expected rise of 10 million new cases (World Health Organisation, 2023). In Australia, it is estimated that approximately 60% of people living with dementia experience behaviours and psychological symptoms of dementia (BPSD), which can include depression, psychosis, and agitation (Australian Institute of Health and Welfare, 2024; Kamoga et al., 2023). BPSD is a major source of caregiver burden and a factor in the decision-making process for admission to residential aged care homes (Chiu et al., 2006), where preventing or treating symptoms early can help alleviate these issues (Kim et al., 2021; Pinyopornpanish et al., 2022).

Antipsychotics are commonly prescribed to assist in the management of BPSD when first-line interventions have failed, with 13% of people living with dementia using these medications in Australia (Lau et al., 2024). However, there is uncertainty about their use in this population due to the limited symptoms they effectively target and their adverse effect profile (Mok et al., 2024; Ohno et al., 2019). A Cochrane review found that there is some evidence that typical antipsychotics may decrease agitation and psychosis, whilst atypical antipsychotics only slightly reduce agitation in people living with dementia (Mühlbauer et al., 2021). However, both drug classes can increase the risk of somnolence, heart failure, stroke and many more adverse events (Mok et al., 2024; Mühlbauer et al., 2021). Therefore, clinicians considering prescribing antipsychotics in people living with dementia should individualise patient assessment of the risks and benefits of therapy (Yunusa et al., 2019). However, a 2021 study revealed that 15.5% of people living with dementia were prescribed an antipsychotic that was considered potentially inappropriate versus 0.8% of those without dementia (Vickers et al., 2021).

With this in mind, it is essential that clinicians are informed with up-to-date and evidence-based information when making decisions about treatment options (Henshall et al., 2019). Clinical decision support tools (CDSTs) are a way of improving prescribing for antipsychotics, which could stratify recommendations according to baseline clinical and demographic characteristics (Henshall et al., 2017). By using a tool that utilises individual patient characteristics, clinicians can customise treatment according to patient needs, a form of personalised care (Henshall et al., 2017). This personalised care could complement clinical decision-making by providing individualised patient information regarding treatment response and adverse outcomes (Kappen et al., 2012). Furthermore, CDSTs can help assist in the shared decision-making process with families and carers improving the awareness and risk of potential side effects (Henshall et al., 2019).

For a CDST to be used in an effective manner, it must incorporate measurable outcomes, patient views and support clinical judgement (Henshall et al., 2017). As part of the development process, the views of potential end users must be explored to ensure CDSTs meet the needs of clinicians and their patients (Garg et al., 2005). To the best of our knowledge, there is no CDST currently available that predicts individual response to antipsychotics and can help with personalised prescribing in people living with dementia in Australia. Investigating this local perspective is important as potential differences in healthcare systems, regulations, prescribing practices, and cultural attitudes may influence the use and perception of CDSTs for antipsychotic prescribing in dementia in Australia. Thus, the aim of this study is to investigate the views of Australian prescribers on the development and use of a digital antipsychotic prescribing support tool in dementia.

## Methods

### Study Design

This qualitative study used semi-structured interviews and followed the Standards for Reporting Qualitative Research (SRQR) (Equator Network, 2023). The study was approved by the University of Sydney Human Research Ethics Committee (2024/HE000171). All participants provided informed consent before the interviews, confirming their full understanding of the study’s purpose, the confidentiality protocols in place, and their right to withdraw at any point. Assent was also obtained at the beginning of the interviews.

### Participant Recruitment

Participants were purposively sampled across a variety of health professions who are directly involved in antipsychotic prescribing in dementia, including geriatricians, psychiatrists, neurologists, general practitioners (GPs), and nurse practitioners (NPs), to gain insights from a range of prescribing clinicians and healthcare settings. NPs in Australia are authorised to prescribe medicines; however, each state and territory has their own legislation which governs NP prescribing (Victoria State Government, 2019). Geographic variability and clinicians’ experience were also considered in the recruitment process. Recruitment of participants was facilitated through flyers, researcher contacts, research networks (Sydney Health Partners Geriatric Medicine Clinical Academic Group, Sydney Dementia Network) and key informants in the area. Furthermore, snowball sampling was used to gather more participants. Invitations were sent to potential participants by ET via email to confirm interest and consent to participate. For their time, participants were also provided a gift card.

### Data Collection

Prior to interviews, participants received a study information sheet, an online consent form, and a brief online survey about demographic characteristics and clinical experience. Consenting clinicians were then interviewed using a semi-structured interview guide (Supplemental table 1). Interview questions were developed from an initial literature review (Tan et al., 2024) and discussions with the research team to elicit perspectives on the following areas: a clinical scenario to ‘set the scene’, factors associated with antipsychotic prescribing, outcomes, patient values and end use.

The interview guide was piloted with one clinician interviewee to ensure questions were appropriately developed to suit the intent of the interview. Online interviews were conducted by ET, an experienced qualitative researcher and pharmacy practice academic, and TT, a qualitative research-trained Pharmacy Honours student, via video call (Zoom Workplace), lasting approximately 30 min. This process was supervised by an experienced qualitative researcher, YJ, who also provided training and support. Interviews were conducted from August to October 2024 and were audio-recorded and transcribed verbatim using Microsoft Word auto transcribing function. Transcripts were verified by TT and imported into QSR Nvivo 14 (Lumivero, 2024) for data management and analysis. Interviews were conducted until data saturation was reached.

### Data Analysis

Transcripts were analysed and initially coded for, by using a set of *a priori* categories structured according to the interview guide by TT. This was followed by thematic analysis using an inductive approach, following the steps outlined by Braun and Clarke (2006), and discussion between TT, ET and YJ. Following initial coding, thematic analysis and its interpretation was used to refine emerging themes by TT, under the supervision of ET and YJ. Any discrepancies were resolved by discussion. Analysis of all transcripts were then finalised in discussion with the wider group of investigators.

## Results

### Participant Characteristics

Fourteen participants were individually interviewed, with six being males. Participants were clinicians that were involved in antipsychotic prescribing in people living with dementia, with the majority being either psychiatrists or geriatricians. Participants worked across a variety of work settings, mainly in hospital and/or residential aged care, with participants most commonly working within Northern Sydney. Participant characteristics are summarised in Table 1.

**Table 1.** Participant Characteristics

Characteristics	Number (%)
Gender	
Male	6 (43)
Age group	
30–39	4 (29)
40–49	3 (21)
50–59	4 (29)
60+	3 (21)
Specialty	
Psychiatrist	4 (29)
Geriatrician	4 (29)
General practitioner	3 (21)
Nurse practitioner	2 (14)
Neurologist	1 (7)
Years of experience	
1–9	3 (21)
10–19	5 (36)
20–29	3 (21)
30–39	2 (14)
40+	1 (7)
Work setting	
Hospital	9 (64)
Public clinic	3 (21)
Private clinic	2 (14)
Residential aged care	4 (29)
Community outreach	2 (14)
General practice	3 (21)
Location of primary practice	
Northern Sydney	6 (43)
Inner West	2 (14)
South Western Sydney	2 (14)
Central coast	2 (14)
Western Sydney	1 (7)
South Eastern Sydney	1 (7)

Themes were organised according to the topic areas about the development and use of the tool: factors for consideration, expected outcomes following antipsychotic use, benefits of using the tool, concerns with using the tool, potential utility, and suggestions for development. Illuminating or example quotes within key themes are presented below.

### Factors for Consideration

*Medical history.* All clinicians recognised the importance of considering the patient’s current medical history prior to prescribing an antipsychotic. They particularly considered comorbidities and concomitant medications due to potential risks of adverse outcomes associated with antipsychotic use. Clinicians also described the importance of frailty when considering prescribing an antipsychotic. Furthermore, clinicians would consider treatment history prior to considering an antipsychotic, and the person’s response.

*Dementia stage or symptom severity*. Clinicians highlighted the importance of considering the stage of dementia or severity of symptoms when determining the need to prescribe an antipsychotic.

*Patient-family considerations.* Caregiver burden was a common factor that clinicians considered prior to prescribing an antipsychotic, either to help the family manage the patient or to prevent institutionalisation and allow the patient to remain at home. Clinicians also expressed the need for consent either by the patient or the carer before antipsychotic use. This would involve discussions of the risks and benefits associated with antipsychotic use as well as patient and/or carer treatment preferences.

Ultimately [it] comes down to a discussion with the family and the patient about risks and benefits, and weighing that up to see how much they’re able to manage the paranoia and non-pharmacological strategies. (Geriatrician 3)

### Expected Outcomes following Antipsychotic Use

*Adverse outcomes*. When prescribing an antipsychotic, clinicians indicated numerous adverse outcomes that they would like to know the risk of when prescribing an antipsychotic to their patients. These could include risk of falls, extrapyramidal side effects, and mortality.

Things from the extreme of an increase in mortality to parkinsonism, extrapyramidal symptoms, falls, difficulties with speech and swallowing, metabolic effects from some antipsychotics. (Neurologist 1)

*Treatment response*. Clinicians highlighted the importance of reducing behaviours of concern and distressing symptoms that the patient is experiencing.

The effectiveness of the medication would be the primary outcome that I would be interested in. (GP 2)

*Outcomes important to the patient and/or their carer*. Clinicians identified that reducing behavioural changes would be an outcome important to the patient and/or their carer. This would allow the patient to continue to live at home independently where possible, ultimately alleviating the burden on the caregiver.

A greater focus on maintaining independence, preserving their autonomy and not being too sedated…that’s important for the patient so they feel they can function. (Psychiatrist 3)

### Benefits of Using the Tool

*Identifying risk*. Clinicians expressed that using the tool could help with patient assessment by identifying potential risks prior to prescribing an antipsychotic. By more explicitly considering risk factors, participants suggested that prescribers might be more cautious prior to prescribing.

It does prompt clinicians to think about the risks of using antipsychotics in this group…if you put in the inputs and you go, OK, there’s quite a high risk if we are going to do it, it might prompt you [to] be much more cautious around the medications. (Psychiatrist 1)

This could be a good thing to say: ‘OK, hold on. Let’s look at what is happening. Let’s look at the risk factors and risk for adverse events or serious adverse events’…I think it’s a good tool to just make clinicians stop a little bit. (NP 1)

Furthermore, some clinicians also communicated that the tool could be used to demonstrate response and risk over time. This could aid in developing a treatment plan based upon the initial output, whilst also assisting in deprescribing if the benefit of using the antipsychotic is minimal or if the risk of adverse events increases.

You could probably use it to see how risk changes with time. So, for example, depending on what the percentages are, as people become more frail, you would expect that that risk would increase with time. And so, it could actually be used as a tool to also deprescribe down the track. (Geriatrician 3)

It also helps with planning treatment ahead of time, like if the tool can tell you all the chances of them falling over. Could we then, before they go home, increase the level of supervision available for these patients and this would be more readily achievable if we have numbers that we can just say ‘this is a percentage chance of falls?’. So, increase the services here to prevent that. (Psychiatrist 2)

*Informed decision-making.* Clinicians communicated that using the tool could help to inform prescribers as well as the patient and/or their carer about antipsychotics. It would also help them to consider more factors than they may usually think about when prescribing an antipsychotic.

Patients, family, as well as the clinician, probably are better informed, especially if it’s not something that they deal with very often. (Geriatrician 3)

More considered, informed decision-making. As I said, people tend to use the same drugs and have limited choices without really thinking through all the factors that should go into a consideration. (Psychiatrist 4)

It might prompt factors that you hadn’t considered into the clinical picture, so that might be of benefit. Or interactions you hadn’t considered. (Psychiatrist 1)

Some clinicians identified that the tool could be used as a reference point for prescribing or a potential way to validate their decision to use an antipsychotic.

I think it’s good. It’s like a kind of template, a starting point and something you can refer to and a validation tool for what they’re on. (GP 1)

Most clinicians expressed that the tool could be used to help in the discussion with the patient and/or their carer about using an antipsychotic. This in turn would assist the informed decision-making process, as the tool could also be used in an educational manner.

It’s a tool for education that you can educate the family, that this is what is, you know, advised. (GP 1)

I think mainly aiding in communication and being able to give families and carers more information. (NP 1)

Additionally, some clinicians suggested it could help in the consenting process for using an antipsychotic, and aid in shared decision-making for treatment goals.

It sort of helps, I guess, both the clinician and the carers and if the patient can participate to sort of understand where the risks sort of lie and whether the benefits are worth pursuing. (Psychiatrist 2)

Clinicians articulated that the tool could help guide the discussion of using antipsychotics by providing more accurate information and giving the patient and/or their carers actual numbers associated with certain outcomes. Clinicians expressed that quantifying the risk would better help inform the shared decision-making process, particularly for patients and their carers who are more data driven.

I think what would be useful is if you could give carers a kind of percentage risk of different outcomes. I think that would be really helpful because people often ask what the percentages are. (Neurologist 1)

Particularly for those patients and their relatives, where they’re a bit more data driven and want…the actual numbers in terms of the possibility of risk, because I tend to mention common side effects, but I don’t always…put a specific number to that. (Psychiatrist 3)

In addition to this, some clinicians voiced that it could also give them more confidence discussing the risks that are associated with antipsychotic use by providing quantified information.

It would give me more confidence that when I’m discussing that with the family or discussing it with the GP or discussing with somebody else, I can kind of make the case to say ‘yes, of course we know there are risks but they’re pretty small’. (NP 1)

It’s hard to pull numbers out of your head…I guess it’s one thing when you just tell them…So, I think it would be quite useful in being able to just give them the numbers. (Psychiatrist 2)

*Potential tool for consistent prescribing practices*. Some clinicians suggested that the tool could potentially help to standardise antipsychotic treatment, whilst promoting a consistent approach to assisting patients in these clinical situations.

It would help sort of standardise certain things as well because I guess psychiatry is done very interestingly, sometimes some people have a method, some people don’t quite have a method, and they tend to stick to things that they’re sort of used to…I think that would probably help them streamline and have a bit more consistent approach to patients rather than sort of relying on, in a way, it’s basically just gut feeling. (Psychiatrist 2)

Other clinicians viewed this tool to have a form of standardised language that allows clinicians and other healthcare professionals to communicate with each other based upon the output of the tool. This would specifically benefit patients who are consulting multiple healthcare professionals.

It can be a form of communication, a bit like, you know, the Heart Foundation, CVD risk factors [where] you plug in the numbers, and you get this result. And that’s something that everyone uses across the board. (GP 1)

I’m a great proponent of standardised tools. While they are not always comprehensive, it gives a clear language if different clinicians are looking at the same person at different times, and we refer quite a lot, or they might be seen by the specialist for something else. (NP 1)

I’ve kind of come across people just being on an antipsychotic and you have to kind of dig back through their notes to see what the rationale was and what was being treated. (Neurologist 1)

In addition, some clinicians recognised the tool as a means to rationalise their decision to prescribe an antipsychotic to their patients. Similarly, it could justify antipsychotic use to prevent delaying treatment in situations where there is hesitation.

I can see why that clinician made that decision or they can see why I made that decision. They may not agree with it, but at least they see where I came from with my decision-making. (NP 1)

Based on what the results show, it may give a clear indication to actually use it rather than delay the use of a treatment for someone who is significantly agitated, which may actually provide them with some benefits. (Geriatrician 3)

### Concerns With Using the Tool

*Complexities of antipsychotic prescribing.* When asked about potential concerns about using the tool, clinicians raised the fact that they would still be guided by patient assessment despite what the output of the tool may be. The clinical decision of using an antipsychotic would be based on situational needs, and this clinical decision would override information from the tool.

I would say that in most cases where someone has bad dementia and there’s significant BPSD, the goals of care are often to focus on comfort and quality of life, and limiting their distressing symptoms. (Geriatrician 1)

I think a lot of the other factors are very much part and parcel of our assessment of the patient, and it’s not something which we would necessarily be challenging or questioning. (Psychiatrist 1)

Moreover, clinicians indicated that if the risks were not presented appropriately, then the output from the tool could adversely affect discussing these issues with the patient and/or their carer. This could potentially delay treatment involving antipsychotic use, where there is clear benefit of its use, as patients could refuse treatment.

I wouldn’t want it to particularly highlight risks of side effects or adverse outcomes at the expense of the patient necessarily agreeing to have the prescription. You know, patients should be fully informed, but particularly with paranoia or psychosis, the more severe cases that I see, there’s obviously very real risks of doing nothing as well. (Psychiatrist 3)

It might actually put yourself, but also the patient and the family, very reluctant to its use and it could delay treatment of something that may be necessary…you’re potentially delaying treatment for something that realistically you would have otherwise started earlier. (Geriatrician 3)

All clinicians were also asked whether the tool could factor in patient and/or carer values. Some clinicians believed that the tool could not, due to the complexity arising from a potential lack of capacity to consent to treatment.

It would be very difficult to really incorporate that as part of your tool. I mean, there may be factors around limited insight challenges with consent and capacity and that’s difficult to quantify within a tool for sure. (Psychiatrist 1)

However, some clinicians did express that, despite the tool’s limitations, it could help guide the discussion surrounding patient values.

I would say that this instrument would be a tool for the clinician to kind of guide that discussion. It wouldn’t necessarily be able to do that role in and of itself in terms of adapting or discussing of values. (Psychiatrist 3)

*Limitations in existing evidence*. The data surrounding the target patient population was a concern to some clinicians, whereby the evidence regarding the use of antipsychotics in people living with dementia may be limited.

We’re not really looking at a choice of antipsychotics. So, any kind of other antipsychotic use is more off label use. There’s probably not a lot of evidence around it. So, I do wonder about the utility of this tool in everyday practice. (Psychiatrist 1)

The other consideration would be whether it’s backed by evidence. (GP 2)

Clinicians further elaborated that the evidence regarding antipsychotic use in this patient population may be poor, as they would not be included in the research of effectiveness.

I would imagine that in terms of research in this population, it would be somewhat limited in the sense that the really aggressive patients probably wouldn’t be enrolled in clinical trials. (Geriatrician 1)

Similarly, there were concerns about how the tool could overestimate antipsychotic risk as the target patient population may already be at high risk of adverse outcomes prior to antipsychotic use.

We are looking at a fairly old and frail population who are already going to be at high risk of a lot of these outcomes…I would wonder whether a lot of it would be attributed to the antipsychotic, when in reality it was the pneumonia or something else not entirely related…it’s hard to say how much of it is just their baseline risk and how much of it is the antipsychotic. (Geriatrician 1)

There may also be conflicting evidence between tools used in clinical practice, where there may be differences in how they quantify certain outcomes that lead to varying outputs.

There are tools to look at anticholinergic burden. However, the tools don’t agree with each other. Different drugs are rated differently because anticholinergic burden is assessed differently. (Psychiatrist 4)

*Potential concerns with technology-based tools.* Many clinicians stressed the importance of utilising clinical judgement in situations where antipsychotics may be clinically indicated, as they communicated that prescribers could become over reliant on using a tool such as this.

The potential concerns are really people’s brains being switched off in trying to make the difficult decisions. (Psychiatrist 4)

I think the thing that I worry about is sometimes if people just go and do like a ticker box thing and don’t actually think about the clinical context. (Geriatrician 4)

The risks I think for them is that you can depend on them completely and stop using your clinical judgement. (NP 2)

Although, other clinicians did not recognise this as a concern, as the tool only provides additional information that should be considered in the clinical decision-making process.

Obviously, you do your own assessment and like any other standardised tool, you still make your own decision. (NP 1)

It’s just an extra layer of information that is easier to access to help us with our clinical decision-making. (Psychiatrist 2)

I wouldn’t say that [the tool] reaches the level of making a recommendation. It’s more just allowing you to do that. (GP 3)

Another issue that clinicians identified was that the tool may be too time consuming, particularly if there were too many inputs needed. This is attributed to the fact that clinicians may be time poor and lack the capacity to use a tool that may be too complex.

I just wonder…in terms of actually getting clinicians to use it. I think clinicians are time poor...I don’t know that they’ll have enough time to go to a website to enter all the details and to get these numbers. (Geriatrician 1)

The user friendliness of the tool, whether it’s easy to use, would be the main consideration for me, whether I’d end up using it or not. If It’s too complicated, then I’d probably shy away from it. (GP 2)

### Potential Utility

*How the clinician would use it.* Clinicians suggested a variety of ways that the tool could be used in practice. It could be used at the point of care with the patient when discussing the use of the antipsychotic, although it would be time dependent.

I would personally probably think about using it at point of care when you’ve got the patient, the family there to both go through it with them there. But I suppose it comes down to time. (Geriatrician 3)

I guess you could also do it with the family at the end of the review and sort of go through, well, these are the options if we were to use a medication like this. (Neurologist 1)

Others proposed that it would be used prior to suggesting an antipsychotic.

I would probably say if I was going out to see somebody and I thought they may need an antipsychotic and I had their medical information and that, I would probably start using it there to give me a broad idea. (NP 1)

*Potential users.* Many clinicians indicated that there could be multiple users, particularly younger practitioners and GPs, as specialists would have less need for the tool, being more familiar with handling these clinical situations.

Look, I’m pretty experienced. And I probably won’t…but I think a lot of GPs would use it and a lot of younger practitioners and non-geriatricians would use it. (Geriatrician 2)

I guess specialists by in large would be more familiar with the side effect profile of these medications and more confident in discussing adverse events…It may be more likely or maybe more valuable in the hands of GPs…they haven’t necessarily had the training or they don’t have the time. (Psychiatrist 3)

Likewise, participants proposed its use for nurses, to assist in the clinical decision-making process for discussions with either the clinician or the family. It could also be used in situations where antipsychotics are prescribed on an as needed basis, reducing unwarranted antipsychotic use.

It would be a useful tool for those nurses to use to then present to the general practitioner and the family. (Geriatrician 2)

Sometimes these medications are PRN as well. So, I guess a registered nurse would probably be making a decision whether it was warranted or not. So, it could maybe be used for that. (Neurologist 1)

Other clinicians advocated the tool’s use for pharmacists who could assist in the safety aspects of using antipsychotics.

Pharmacists can say ‘oh, you know you put in this but according to my calculation’…they can use it as well to help with safety. (GP 1)

So, during the sort of medication review they [pharmacists] would use the tool and then convey that information to the GP. (Geriatrician 2)

### Suggestions for Development

For the development of the tool, clinicians would like to see increased accessibility through a cloud-based service or an app that could be used on their mobile phone. Similarly, they advised that the tool should be visually aesthetic, whilst also smooth to use. Additionally, they would like to have its data continuously updated as evidence and guidelines change over time to ensure its validity.

I think you just want to make it relatively frictionless and easy to use, so it’s picked up. I think if it becomes challenging…then people will just stop using it. (Psychiatrist 3)

Most clinicians suggested that prompts to aid in decision-making would be helpful, including the type of antipsychotic used and comparative information in terms of efficacy and safety. This may include an explanation of what the output of the tool means, whilst also having understandable information displays to discuss use of antipsychotics with the patient and/or their carer.

You could think just in terms of data display, how best to display that for the benefit of the patient or the relative so they could kind of better understand it…[to] augment or help the clinician with their usual kind of verbal explanation of the risks and benefits. (Psychiatrist 3)

In fact, the most helpful thing I think would be if you could provide a matrix of the commonly used antipsychotics…then having the matrix show relative risks…you could see which ones are probably better to use. (GP 3)

## Discussion

This qualitative study highlights the overall positive perception of clinicians in using a digital tool to assist with antipsychotic prescribing in dementia but identifies potential concerns and issues that must be considered when developing the tool for practice.

Clinicians highlighted several factors for consideration prior to prescribing an antipsychotic for BPSD, including medical history, dementia stage or symptom severity, and patient-family considerations. Similarly, several studies and clinical guidelines have also identified these factors as essential considerations for antipsychotic prescribing (Cognitive Decline Partnership Centre, 2016; Dennis et al., 2017; National Institute for Health and Care Excellence, 2018; Rogowska et al., 2023; Tan et al., 2024).

In terms of ‘expected outcomes following antipsychotic use’, participants expressed that they would like to see a reduction in the behaviours of concern, allowing patients to remain at home. However, they would monitor for adverse effects that come with antipsychotic use. These opinions are consistent with Yunusa et al. (2019) where a balance is needed between efficacy and safety of using antipsychotics in people living with dementia. In the context of CDSTs for antipsychotic prescribing, our findings expand on previous research by focusing on the BPSD context, where goals of care and care contexts often differ from other psychiatric disorders. This distinction underscores the need for novel tools that can address the complexities of dementia care.

Clinicians highlighted several ‘benefits of using the tool’, including identifying risk and aiding informed decision-making. This is consistent with similar studies exploring use of CDSTs (Henshall et al., 2019; Manski-Nankervis et al., 2020), where clinicians would be more aware of the potential risk of side effects, whilst also helping to inform their decision to use certain medications. Furthermore, Henshall et al. (2019) emphasises the role CDSTs can play in clinical practice where it could be used as a monitoring and review tool for long-term use of antipsychotics, reiterating the findings of our study. Similarly, our study highlights how CDSTs can be used as an educational tool by the patient and/or their carer. Henshall et al. (2019) and Manski-Nankervis et al. (2020) also found that CDSTs can promote informed choice, provide evidence-based information, and can be used to open discussions between clinicians and the patient regarding treatment plans and direction. However, Müller et al. (2023) found that decision aids like CDSTs can only be used as a basis for discussion for prescribing medication; shared decision-making is still essential in this process. Our study also found that the tool could support consistent prescribing practice. Similarly, Henshall et al. (2017) found that clinicians viewed CDSTs as a way to standardise prescribing practices, as preferences for certain medicines were not always informed by the latest evidence, but previous experience. The idea of a standardised language across multidisciplinary team settings was raised in our study; however, Sheehan et al. (2013) posed that there are barriers to standardised language and interdisciplinary communication when using CDSTs due to variations in styles of practice, where clinicians could input varying information resulting in different outputs of the tool. Therefore, this needs to be considered in the future development of the tool.

Clinicians discussed ‘potential utility’ of the tool, especially by younger practitioners and GPs. This is consistent with previous literature on implementation of CDSTs for antibiotic prescribing, where benefit was seen in more junior doctors who had limited experience with clinical guidelines, although more experienced GPs did identify its benefit in their own practice with regard to communication with the patient (Manski-Nankervis et al., 2020). Furthermore, previous literature has found that nurses influence prescribing decisions made by clinicians, including when prescribing antipsychotics for delirium (Jutel & Menkes, 2010; Tomlinson et al., 2021). As a result, the tool could be used to help nurses communicate with clinicians on decisions to prescribe antipsychotics in people living with dementia, as identified in our study. Moreover, pharmacists using the tool was a prominent idea raised and is consistent with a previous study showing CDSTs can support hospital pharmacists to optimise medication therapy (Yan et al., 2021). In the context of BPSD management, our findings offer new insights into the broad range of interdisciplinary end users, particular junior clinicians and non-specialists, for CDSTs in antipsychotic prescribing.

The findings also highlight potential barriers for use of the tool. Many clinicians highlighted that antipsychotic prescribing is complex, requiring prescribers to use their clinical judgement in determining risk versus benefit, despite what a CDST may suggest. For example, previous studies have identified that an anticipation of aggressive behaviours and a need to protect staff and family members from harm, may influence antipsychotic prescribing, where potential for physical harm far outweighs the possible risk of adverse events caused by the medication (Tomlinson et al., 2021; Walsh et al., 2018). Our results also indicate how the tool could influence patient’s’ decision and potentially delay beneficial treatment. Henshall et al. (2017) found that receiving excessive information regarding the side effect profile of medication can cause unnecessary concern for the patient and carer; however, participants felt that they did not need to be aware of every detail, as long as the prescriber was well-informed about the treatment. Additionally, this study highlights how clinicians are aware of the lack of evidence of antipsychotic efficacy in people living with dementia, confirmed by previous studies (Macfarlane & Cunningham, 2021; Rogowska et al., 2023), and therefore this should be considered when developing the tool. Likewise, clinicians indicated that prescribers may potentially void their clinical judgement and over-rely on the tool. This has been substantiated in previous work, where there was an identified relationship between a user’s trust in a CDST and their reliance on its use (Bussone et al., 2015).

The findings provide suggestions for the usability of the tool in practice. Accessibility through an app or a cloud-based service was suggested by several participants, and is supported by previous literature (Henshall et al., 2019; Müller et al., 2023). Manski-Nankervis et al. (2020) identified that integration of the CDST into GP practice software reduced time wasted and frustration for GPs in accessing clinical resources, which could be considered for the development of the tool. Additionally, our study indicated that clinicians would like to see design layouts and explanations to help themselves and the patient and family to understand the effectiveness and risks of antipsychotic medications. This is similar to previous literature where a display of the ‘best’ drugs for certain indications with risk estimates of adverse outcomes was viewed positively (Müller et al., 2023). Interestingly, Manski-Nankervis et al. (2020) found that GPs were more inclined to use recommendations that the CDST had outputted if there was a direct link to the relevant guideline.

### Clinical Implications

The need to reduce inappropriate prescribing of antipsychotics in people living with dementia is imperative (Lau et al., 2024), with only 10% of antipsychotics prescribed to this population deemed appropriate (Macfarlane & Cunningham, 2021). The Royal Commission into Aged Care Quality and Safety found evidence of overprescribing of psychotropics and lack of efficacy in older adults (Aged Care Quality and Safety Commission, 2022). Thus, the findings of this study can help inform the development of a tool that has potential to reduce inappropriate prescribing of antipsychotics. Additionally, some clinician barriers to optimising prescribing in people living with dementia, including a lack of data availability, difficulty in assessing efficacy and safety, and patient/caregiver concerns (Green et al., 2019), can be addressed. By culminating the evidence on antipsychotic use into one accessible tool, better informed decision-making for clinicians, patients and caregivers can be achieved.

### Strengths and Limitations

This study has several strengths and limitations. This study was strengthened by the inclusion of a variety of clinicians that prescribe antipsychotics to people living with dementia, providing insights on the potential benefit and concerns of the tool. Whilst the sample size of this study is small, we ensured data saturation was reached by recruiting two more participants. This study also used a thematic analysis approach which allows researchers to summarise and interpret multiple data sets despite a small sample size, whilst enabling an understanding of thoughts and experiences of an individual (Clarke & Braun, 2017; Kiger & Varpio, 2020), aligning with this study’s aims.

However, there are some limitations. Firstly, participants were predominantly sampled through direct contacts of researchers and snowballing. This may have introduced selection bias towards more knowledgeable and interested clinicians. To minimise selection bias, we purposively sampled clinicians from both metropolitan and regional areas of New South Wales, while also considering clinician experience, with participants ranging from junior to senior clinicians. Moreover, with only one researcher verifying audio recordings of transcriptions produced by technology, there may be potential inaccuracies in the transcripts that were analysed. While this study focused on the views of prescribers, it would be important for future research to also investigate the perspectives of patients, carers and non-prescribers such as pharmacists and nurses.

## Conclusion

Our study found a range of considerations in the development and implementation of the tool in practice, providing insights for future use. As such, there are several benefits of using the tool, particularly in identifying risk associated with antipsychotic use and aiding informed decision-making between all parties. Its potential utility in practice not only lies with the prescriber but extends to the broader healthcare team. Though the tool was generally positively received, potential concerns and suggestions expressed by clinicians should be considered in any future development. Building on the findings of this study, next steps will focus on co-designing the tool with input from clinicians, patients, and carers to ensure it meets the needs of all stakeholders.

## Supplemental Material

Supplemental Material - Developing a Novel Digital Tool for Personalised Antipsychotic Prescribing in People Living With Dementia: The Views of Australian Clinicians Supplemental Material for Developing a Novel Digital Tool for Personalised Antipsychotic Prescribing in People Living With Dementia: The Views of Australian Clinicians by Timothy Josh D. Tan, Yun-Hee Jeon, Edward C. Y. Lau, Sarah N. Hilmer, Lee-Fay Low, Christine Y. Lu, Edwin C. K. Tan in Dementia
