# Supplementary material for: Developing a Novel Digital Tool for Personalised Antipsychotic Prescribing in People Living With Dementia: The Views of Australian Clinicians
Source: Dementia (London). 2025 Aug 5;25(6):1191–209. doi: 10.1177/14713012251366757 (PMC13304906; doi:10.1177/14713012251366757)
Supplement: Supplemental Material - Developing a Novel Digital Tool for Personalised Antipsychotic Prescribing in People Living With Dementia: The Views of Australian Clinicians [file sj-pdf-1-dem-10.1177_14713012251366757.pdf]

Supplementary table 1. Interview guide

Clinical scenario[1]:

Case: A 65-year-old woman with early onset dementia was cared for at home by her husband. She was becoming very paranoid that her husband was having an affair and was going to leave her. She attempted to lock her husband in so he could not leave. The husband and family did not want her institutionalised but were at their wit's end. A trial of SSRIs was unsuccessful.

- How would you approach this? What strategies would you use to manage this?
- How do you make a decision to use an antipsychotic in this scenario?

Outcomes to be measured

- What are the main outcomes you want to see from prescribing an antipsychotic?
- What outcomes do you think are important to your patient/their carer?
    - Prompts
        - Effectiveness (BPSD, psychosis, agitation)
        - Safety (sedation, falls, cognitive worsening, cerebrovascular disease, mortality)
        - Other (quality of life, carer burden etc)

Factors to be considered when prescribing

- What do you think of these factors? Do you think they are all relevant?
    - Age
    - Sex
    - Comorbidities (e.g. cerebrovascular disease)
    - Concomitant medications (e.g. benzodiazepines, antidepressants)
    - Dementia subtype
    - BPSD symptoms
    - Antipsychotic (type, dose)
- Are there any other ones that you think are important to consider?

Patient values

- Patient preferences and values are important to consider when prescribing antipsychotics. How can the tool take this into consideration?

---

[1] This scenario was adapted from Levenson, S. (2024). Antipsychotics in Perspective: Past, Present, and Future (Article 2 of 3). Journal of the American Medical Directors Association, 25(2), 183-188.e187. https://doi.org/10.1016/j.jamda.2023.11.007

End use

- Can you see yourself using this tool in your practice? How do you see yourself using this tool in your practice?
    - How would it fit/be integrated into your workflow/clinical practice?
- What do you see as potential concerns with this tool?
- What do you see as potential benefits?
- Have you come across any similar risk assessment tools or calculators like this? What are your thoughts on these?
- Do you have any other comments about the development of this tool?

BPSD – behaviours and psychological symptoms of dementia
